# Supplementary material for: Diversification of Bourbon Virus in New York State
Source: Microorganisms. 2023 Jun 15;11(6):1590. doi: 10.3390/microorganisms11061590 (PMC10304581; doi:10.3390/microorganisms11061590)
Supplement: Supplementary file 1 [file microorganisms-11-01590-s001.zip › microorganisms-2423036-supplementary.pdf]

microorganisms

MDPI

Supplementary Materials

Table S1. Primer sets used for whole genome sequencing of recent Bourbon virus (BRBV) strains from New York State.

Primer	Upstream/downstream primer sequence (5′-3′)	Target
BRBV-S6-20	CGCTTCACAAGAGAAATGGC	Segment 6- M
BRBV-S6-868c	TGTAGAGGTTGTCTCTGATGAT	Segment 6- M
BRBV-S5-13	CAGTTGTTCAACATGCAGTC	Segment 5- NP
BRBV-S5-1398c	CAGAGCCAGGCCTATGCCTCG	Segment 5- NP
BRBV-S4-12	GCAGATTGTCCAAAGATGAAT	Segment 4- GP
BRBV-S4-1548c	TCTTAATAGACCCGCCTGCA	Segment 4- GP
BRBV-S3-13	CAGTAATTGTCAATCATGGA	Segment 3- PA
BRBV-S3-1919c	CTACATATAAACATTTGGCA	Segment 3- PA
BRBV-S2-11	AGCAGTTGTTAACCATGAATCT	Segment 2- PB1
BRBV-S2-2152c	ACTAACCCAACTCCTCAACCAG	Segment 2- PB1
BRBV-S1-24	CATACGCACAGATGGAGGGCA	Segment 1- PB2
BRBV-S1-1483c	TAGTTGGTGAGTTGCTGCATAC	Segment 1- PB2
BRBV-S1-1238	GACATTCTGTACTACACAGCATC	Segment 1- PB2
BRBV-S1-2308c	CTACTTCCAGGAAGGTCCG	Segment 1- PB2

Table S2. Bourbon virus (BRBV) strains used in mutational analysis and phylogenetic trees including New York State BRBV strains, midwestern BRBV strains, and distantly related orthomyxoviruses Batken, Dhori, Oz, and Thogoto viruses.

Strain Designation	Location	Year	Source	Accession Number
BRBV NY21-1814	Suffolk County, NYS	2021	*A. americanum* pool	OQ513912, OQ513913, OQ513914, OQ513915, OQ513916, OQ513917
BRBV NY21-2143	Suffolk County, NYS	2021	*A. americanum* pool	OQ513918, OQ513919, OQ513920, OQ513921, OQ513922, OQ513923
BRBV NY21-2666	Suffolk County, NYS	2021	*A. americanum* pool	OQ513924, OQ513925, OQ513926, OQ513927, OQ513928, OQ513929
BRBV Original	Bourbon County, MO	2014	Human	KP657750.3, KP657749.3, KU708255.1, KP657748.2, KU708254.2, KU708253.2
BRBV STL	St. Louis, MO	2017	Human	MK453529.1, MK453528.1, MK453527.1, MK453526.1, MK453525.1, MK453524.1
BRBV MO-2013-2499	Gentry County, MO	2013	*A. americanum* pool	MH880287.1, MH880288.1, MH880289.1, MH880290.1, MH880291.1, MH880292.1
Batken Virus	Kyrgyzstan	?	*Hylamma spp.*	MT628416.1, MT628417.1, MT628418.1, MT628419.1, MT628420.1, MT628421.1
Dhori Virus	?	?	?	NC_034261.1, NC_034263.1, NC_034254.1, NC_034255.1, NC_034262.1, NC_034256.1
Oz Virus	Ehime, Japan	2013	*A. testudinarium* pool	NC_040730.1, NC_040731.1, NC_040732.1, NC_040735.1, NC_040733.1, NC_040734.1
Thogoto Virus	?	?	?	NC_006508.1, NC_006495.1, NC_006496.1, NC_006506.1, NC_006507.1, NC_006504.1
